# Data regarding the growth of *Lactobacillus acidophilus* NCFM on different carbohydrates and recombinant production of elongation factor G and pyruvate kinase

**DOI:** 10.1016/j.dib.2017.07.021

**Published:** 2017-07-14

**Authors:** Hasan Ufuk Celebioglu, Sita Vaag Olesen, Kennie Prehn, Sampo J. Lahtinen, Susanne Brix, Maher Abou Hachem, Birte Svensson

**Affiliations:** a Enzyme and Protein Chemistry, Department of Biotechnology and Biomedicine, Technical University of Denmark, Denmark; b Active Nutrition, DuPont, Nutrition & Health, Finland; c Disease Systems Immunology, Department of Biotechnology and Biomedicine, Technical University of Denmark, Denmark; d Protein Glycoscience and Biotechnology, Department of Biotechnology and Biomedicine, Technical University of Denmark, Denmark

## Abstract

The present study describes the growth of the very well-known probiotic bacterium *Lactobacillus acidophilus* NCFM on different carbohydrates. Furthermore, recombinant production of putative moonlighting proteins elongation factor G and pyruvate kinase from this bacterium is described. For further and detailed interpretation of the data presented here, please see the research article “Mucin- and carbohydrate-stimulated adhesion and subproteome changes of the probiotic bacterium *Lactobacillus acidophilus* NCFM” (Celebioglu et al., 2017) [1].

**Specifications Table**

**Table:** 

Subject area	*Biology*
More specific subject area	*Microbiology, Biochemistry*
Type of data	*Table, graph, figure*
How data was acquired	*Bacterial growth in batch cultures, Heterologous production of recombinant proteins*
Data format	*Raw and analyzed*
Experimental factors	*Bacterial cells were grown on different carbohydrates until stationary phase (24 h) in batch cultures. Genes encoding elongation factor G and pyruvate kinase were cloned and recombinantly produced in Escherichia coli cells.*
Experimental features	*Growth was measured at 600 nm at the stationary phase (24 h). Recombinant elongation factor G and pyruvate kinase were purified using HisPur™ Cobalt Purification Kit (Thermo Scientific), followed by SDS-PAGE to visualize the purified proteins.*
Data source location	*Technical University of Denmark, Kgs. Lyngby, Denmark*
Data accessibility	*All data are presented in this article*

**Value of the data**

• Growth data presented here shows growth potential of the probiotic bacterium *Lactobacillus acidophilus* NCFM on different carbon sources.
• The growth data may be used by researchers to understand the ability of the bacterium to grow on different carbon sources.
• Data regarding recombinant production of elongation factor G and pyruvate kinase from *Lactobacillus acidophilus* NCFM may be used by researchers to apply the same procedure.
• Data shown here and in Ref. [1] are useful for the researchers who are working on gut microbiota, probiotic bacteria, carbohydrate-microbe interactions, and moonlighting proteins.

## 1 Data

**Fig. 1:**
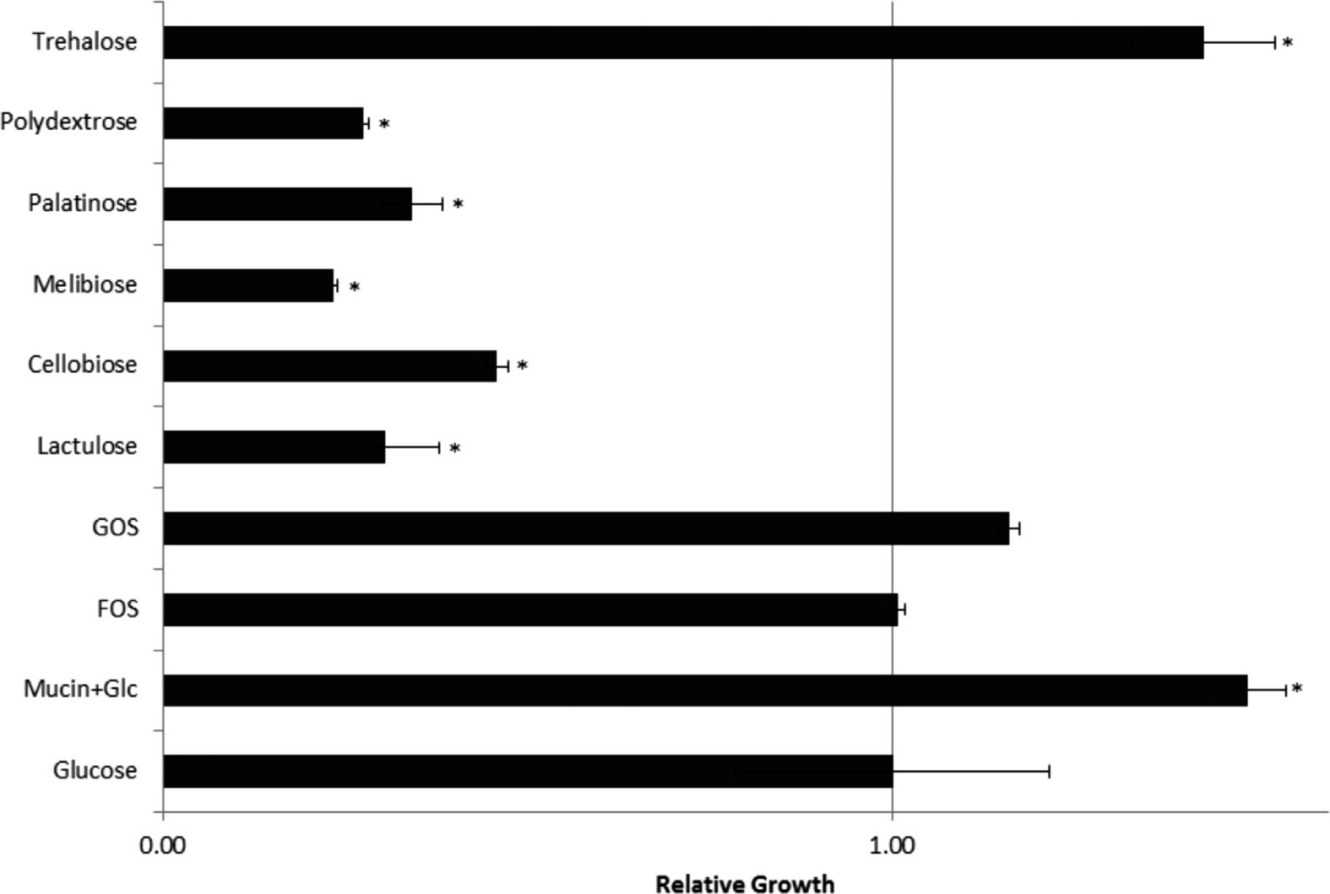
*in vitro* evaluation of growth of *Lactobacillus acidophilus* NCFM (early stationary phase, 24 h) on different carbon sources (1%) or supplemented with mucin (0.1%). Asterisk (*) indicates that the difference in growth of the bacteria are statistically significant compared to growth on glucose (*p* ≤ 0.05).

**Fig. 2:**
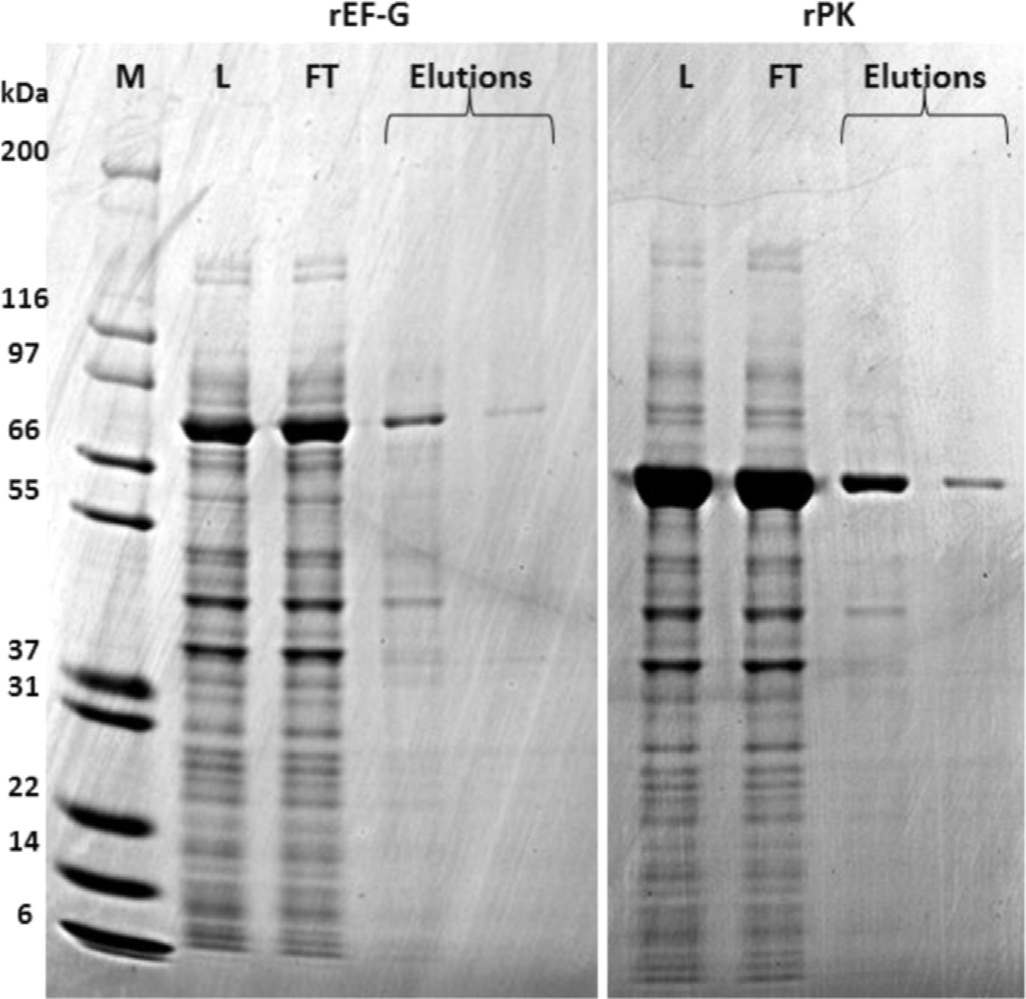
SDS-PAGE of purified recombinant elongation factor G (rEF-G) and pyruvate kinase (rPK). M, molecular weight marker; L, lysate of *E. coli* BL21(DE3); FT, flow-through; Elutions, eluted proteins from HisPur Cobalt resin.

The extensively used probiotic bacterium *Lactobacillus acidophilus* NCFM was grown on nine different carbohydrates including growth on glucose supplemented with mucin (Fig. 1). The putative moonlighting proteins elongation factor G and pyruvate kinase also identified from this bacterium by differential proteomics [1] were recombinantly produced using the primers shown in Table 1 and purified by his-tag affinity chromatography. The purified proteins were analysed by SDS-PAGE (Fig. 2). Table 2 reports on the mass spectrometric identification of the two purified proteins without any identification of *E. coli* proteins.

**Table 1:** Primers used for cloning of *lba0289* encoding elongation factor G and *lba0957* encoding pyruvate kinase.

**Gene**	**Primers**
*lba0289* (Elongation factor G)	**F:**CGCGCGGCAGCCATATGAGGAGAGACTAATTTATGGCTAACA **R:**GCTCGAATTCGGATCCTTATTCAGCGTCGCCG
*lba0957* (Pyruvate kinase)	**F:**CGCGCGGCAGCCATATGGAGAGGATTTATTAAATAATGAAGAAAACT **R:**GCTCGAATTCGGATCCTTAAAGGTTTGAGATTTCACCTTG

**F:** Forward Primer

**R:** Reverse Primer

**Table 2:** MALDI-TOF MS results of recombinantly produced elongation factor G and pyruvate kinase of *Lactobacillus acidophilus* NCFM. No *E. coli* proteins were identified.

**Protein name**	**Database**	**Accession number**	**Score**	**Expect**	**Mw/pI**	**Peptides matched/identified**	**Protein sequence coverage**
**rEF-G**	NCBIprot	YP_193213.1	173	7.7e-11	76,806/4.94	42/169	55%
**rPK**	NCBIprot	YP_193840.1	231	1.2e-16	63,136/5.23	39/145	62%

## 2 Experimental design, materials and methods

*L. acidophilus* NCFM (NCFM) (1.50 × 10^10^ CFU/g DuPont, USA Inc., Madison, US) was grown aerobically without shaking at 37 °C in batch cultures (50 mL) in semisynthetic lactic acid bacteria medium (LABSEM) [2] containing 1% FOS (Sigma-Aldrich); GOS; polydextrose (both DuPont); melibiose (Fluka); lactulose (Sigma-Aldrich); cellobiose (Sigma-Aldrich); isomaltulose (palatinose; Sigma-Aldrich); or trehalose (Sigma-Aldrich); and the reference glucose (Sigma-Aldrich). Porcine gastric mucin to 0.1% (Sigma-Aldrich) was included in cultures with 1% glucose. The bacterium was sub-cultured for three cycles in LABSEM and growth was monitored at early stationary phase (24 h) by measuring absorbance at 600 nm.

Gene-specific primers for *lba0289* (elongation factor G, EF-G) and *lba0957* (pyruvate kinase, PK) with extra 15 bp complementary to the pET28a(+) vector linearized with BamHI and NdeI (Table 1) were designed using CLC Main Workbench software (Qiagen), primer blasted (NCBI), and used to amplify genes by PCR. Cloning was performed with In-Fusion Cloning kit (Clontech) *per* the user manual. The resulting plasmids were transformed into competent *E. coli* DH5α and positive colonies were selected using kanamycin LB agars. Inserted genes were confirmed by sequencing (GATC Biotech). Soluble recombinant proteins were obtained in *E. coli* BL21 (DE3) induced with 0.1 mM isopropyl-β-D-thiogalactoside. Cells were disrupted using a high-pressure cell homogenizer (Stanstead), followed by centrifugation (10,000 × *g*, 20 min). Recombinant proteins (rEF-G and rPK) were purified (HisPur™ Cobalt Purification Kit; Thermo Scientific) according to the manufacturer’s instructions and verified by SDS-PAGE (Fig. 2) [3].

Bands corresponding to elutions in Fig. 2 were excised manually, subjected to in-gel degradation by trypsin and MS protein identification. Briefly, gel pieces were washed with 40% ethanol (200 µL, 10 min) followed by acetonitrile (ACN) (50 µL), reduced with DTT (10 mM in 100 mM NH_4_HCO) and alkylated with iodoacetamide (55 mM in 100 mM NH_4_HCO), incubated with 12.5 ng/mL trypsin (Promega) in 10 mM ammonium bicarbonate (5 µL, on ice, 45 min), added 10 mM ammonium bicarbonate (10 µL), and incubated (37 °C, overnight). Supernatant (1 µL) was applied onto an Anchor Chip target (Bruker-Daltonics), added matrix (1 µL 0.5 mg/mL CHCA in 90% ACN, 0.1% TFA) and washed (2 µL 0.02% TFA). MS spectra were obtained using an Ultraflex II MALDI-TOF MS mass spectrometer (Bruker-Daltonics) in auto-mode with Flex Control v3.0 (Bruker-Daltonics) and processed by Flex Analysis v3.0 (Bruker-Daltonics). Spectra were externally calibrated by trypsin-generated β-lactoglobulin peptides (5 pmol/mL). MS spectra were searched against the NCBIprot database (ver. 20170215) or SwissProt for bacteria using the MASCOT 2.0 software (http://www.matrixscience.com) integrated with BioTools v3.1 (Bruker-Daltonics). Protein identifications by Peptide Mass Fingerprinting (PMF) were confirmed with a MASCOT score of 80 (60 for SwissProt), *p* ≤ 0.05 and a minimum of six matched peptides.
