# Supplementary material for: Data regarding the growth of Lactobacillus acidophilus NCFM on different carbohydrates and recombinant production of elongation factor G and pyruvate kinase
Source: Data Brief. 2017 Jul 14;14:118–22. doi: 10.1016/j.dib.2017.07.021 (PMC5567391; doi:10.1016/j.dib.2017.07.021)

**Supplementary Figure S1** *In vitro* evaluation of growth of *Lactobacillus acidophilus* NCFM (early stationary phase, 24 h) on different carbon sources (1%) or supplemented with mucin (0.1%). Asterisk (*) indicates that the difference in growth of the bacteria are statistically significant compared to growth on glucose (p≤0.05).


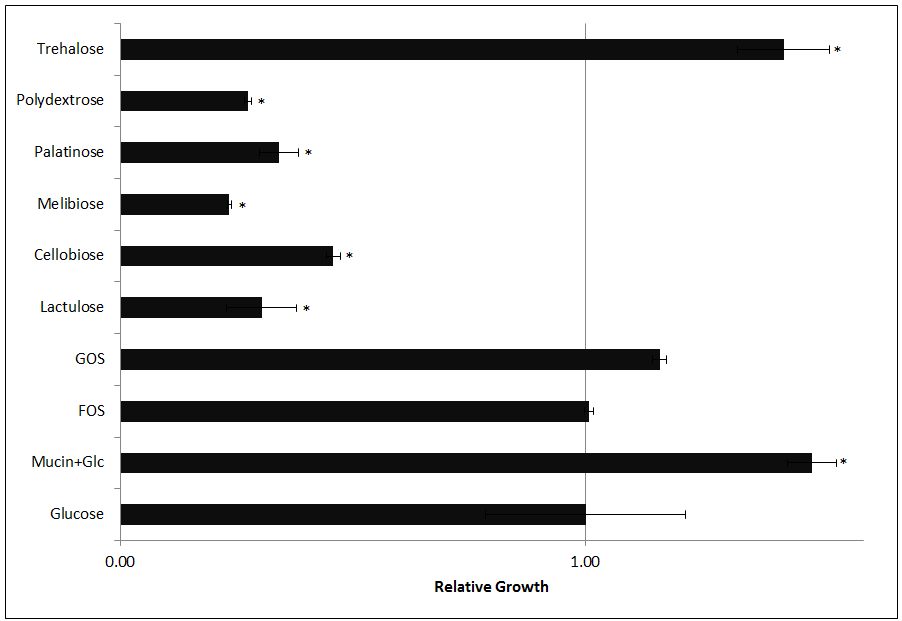

Supplement: Supplementary file 1 — Supplementary material [file mmc1.docx]
