# Supplementary material for: Data regarding the growth of Lactobacillus acidophilus NCFM on different carbohydrates and recombinant production of elongation factor G and pyruvate kinase
Source: Data Brief. 2017 Jul 14;14:118–22. doi: 10.1016/j.dib.2017.07.021 (PMC5567391; doi:10.1016/j.dib.2017.07.021)

**Supplementary Figure S2:** SDS-PAGE of purified recombinant elongation factor G (rEF-G) and pyruvate kinase (rPK). M, molecular weight marker; L, lysate of *E.coli* BL21(DE3); FT, flow-through; Elutions, eluted proteins from HisPur Cobalt resin.


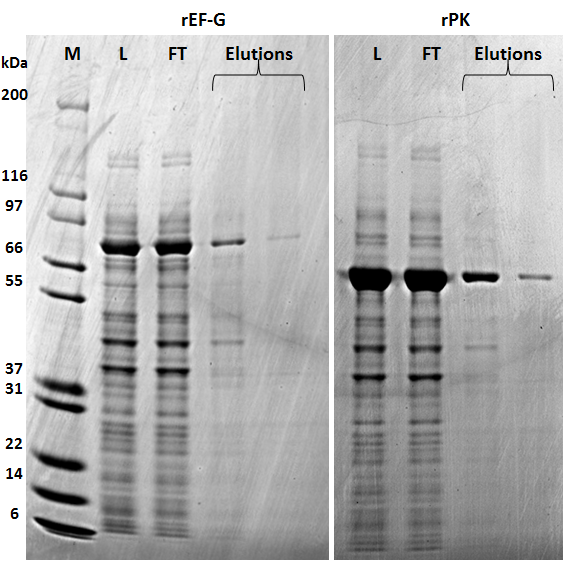

Supplement: Supplementary file 2 — Supplementary material [file mmc2.docx]
