# Supplementary material for: Data regarding the growth of Lactobacillus acidophilus NCFM on different carbohydrates and recombinant production of elongation factor G and pyruvate kinase
Source: Data Brief. 2017 Jul 14;14:118–22. doi: 10.1016/j.dib.2017.07.021 (PMC5567391; doi:10.1016/j.dib.2017.07.021)
Supplement: Supplementary file 3 — Supplementary material [file mmc3.docx]

**Supplementary Table S1:** Primers used for cloning of *lba0289* encoding elongation factor G and *lba0957* encoding pyruvate kinase.

| **Gene** | **Primers** |
| --- | --- |
| *lba0289*  (Elongation factor G) | **F:**CGCGCGGCAGCCATATGAGGAGAGACTAATTTATGGCTAACA  **R:**GCTCGAATTCGGATCCTTATTCAGCGTCGCCG |
| *lba 0957*  (Pyruvate kinase) | **F:**CGCGCGGCAGCCATATGGAGAGGATTTATTAAATAATGAAGAAAACT  **R:**GCTCGAATTCGGATCCTTAAAGGTTTGAGATTTCACCTTG |

**F:** Forward Primer

**R:** Reverse Primer
